# Diversity and Noise Effects in a Model of Homeostatic Regulation of the Sleep-Wake Cycle

**DOI:** 10.1371/journal.pcbi.1002650

**Published:** 2012-08-23

**Authors:** Marco Patriarca, Svetlana Postnova, Hans A. Braun, Emilio Hernández-García, Raúl Toral

**Affiliations:** 1 IFISC, Instituto de Física Interdisciplinar y Sistemas Complejos (CSIC-UIB), Campus Universitat de les Illes Balears, Palma de Mallorca, Spain; 2 National Institute of Chemical Physics and Biophysics, Tallinn, Estonia; 3 School of Physics, The University of Sydney, Sydney, New South Wales, Australia; 4 Center for Integrated Research and Understanding of Sleep, The University of Sydney, Sydney, New South Wales, Australia; 5 Neurodynamics Group, Physiology Institute, Marburg University, Marburg, Germany; Indiana University, United States of America

## Abstract

Recent advances in sleep neurobiology have allowed development of physiologically based mathematical models of sleep regulation that account for the neuronal dynamics responsible for the regulation of sleep-wake cycles and allow detailed examination of the underlying mechanisms. Neuronal systems in general, and those involved in sleep regulation in particular, are noisy and heterogeneous by their nature. It has been shown in various systems that certain levels of noise and diversity can significantly improve signal encoding. However, these phenomena, especially the effects of diversity, are rarely considered in the models of sleep regulation. The present paper is focused on a neuron-based physiologically motivated model of sleep-wake cycles that proposes a novel mechanism of the homeostatic regulation of sleep based on the dynamics of a wake-promoting neuropeptide orexin. Here this model is generalized by the introduction of intrinsic diversity and noise in the orexin-producing neurons, in order to study the effect of their presence on the sleep-wake cycle. A simple quantitative measure of the quality of a sleep-wake cycle is introduced and used to systematically study the generalized model for different levels of noise and diversity. The model is shown to exhibit a clear diversity-induced resonance: that is, the best wake-sleep cycle turns out to correspond to an intermediate level of diversity at the synapses of the orexin-producing neurons. On the other hand, only a mild evidence of stochastic resonance is found, when the level of noise is varied. These results show that disorder, especially in the form of quenched diversity, can be a key-element for an efficient or optimal functioning of the homeostatic regulation of the sleep-wake cycle. Furthermore, this study provides an example of a constructive role of diversity in a neuronal system that can be extended beyond the system studied here.

## Author Summary

All biological systems are inherently noisy and heterogeneous. Disorder is mostly expected to disturb proper functioning of a system, like it can be the case with noise in a radio signal. However, it has been demonstrated by numerous studies that noise can actually improve signal encoding – the so-called stochastic resonance phenomenon. Recently, it was discovered that quenched diversity (heterogeneity) can also enhance the response of a system to an external perturbation (diversity-induced resonance). In this study we investigate the role of noise and diversity in a neuronal model of sleep-wake cycles based on the dynamics of the wake-promoting orexin neurons that is crucial for stability of wake and sleep states. We demonstrate that suitable levels of diversity introduced in the orexin neurons can significantly improve the quality of the sleep-wake cycle, and may be essential for proper sleep-wake periodicity. Noise, on the other hand, provides only a mild improvement.

## Introduction

Disorder, which originates from both noise and diversity, is naturally present in all biological systems. In neuronal systems some examples are the random opening and closing of ion channels, the multitude of stochastic input currents in the neurons, and the diversity of shapes, sizes, and electrophysiological properties of the neurons [1], [2]. Disorder is often considered to be harmful to the systems' functioning and to information encoding. However, it was likewise repeatedly demonstrated that a certain level of disorder can facilitate signal encoding by enhancing system's response to an external stimuli. For instance, quenched diversity clearly shows its constructive role in the phenomenon of diversity-induced resonance, in which an assembly of heterogeneous excitable units presents an optimal response to an external forcing for a suitable intermediate degree of heterogeneity [3], [4], [5]. Similar constructive effects can be observed in the presence of noise. For example, interplay of noise and nonlinear forces produces the directed motion of motor proteins [6], order-disorder transitions, oscillations, and synchronization in assemblies of excitable units [7], [8], [9], and an optimized system response in the ubiquitous phenomenon of stochastic resonance [10], [11], e.g. in ion-channels and neurons [12], [13], [14], [15], [16], [17].

In the present study we examine the effects of noise and diversity (heterogeneity) in a physiologically based neuronal model of sleep-wake cycles [18]. This model introduces a novel mechanism of the homeostatic regulation of sleep based on the dynamics of a wake-promoting neuropeptide orexin (also called hypocretin), assuming depression of orexinergic synapses during wakefulness and their recovery during sleep. This mechanism is based on the experimental findings of the essential role of orexin system in maintaining wakefulness and its ability to integrate the sleep-wake relevant information coming from many brain areas [19], [20] and respond to changes in the body external and internal environments by encoding the body activity state, energy balance, sensory and emotional stimuli [21], [22].

In the original model interaction between only two representative neurons is simulated: the orexin neuron and the local glutamate neuron that are reciprocally connected to each other according to the experimentally established physiological connections [23]. Both orexin and glutamate neurons are firing during wakefulness and are silent during sleep. The transitions between firing and silence are governed by the interplay between the circadian input and homeostatic mechanisms as initially proposed by Borbely [24]. For simplicity, in this model only a single type of orexin neurotransmitter (instead of the two types actually known) is considered, and it is assumed that the system can be either in the wake state or in a generic non-Rapid Eye Movement sleep state, without specifying ultradian structure of sleep. Also this model did not consider noise effects, and diversity could not be included since there are only two neurons present.

In the present paper we extend the above described two-neuron model to a more realistic multi-unit model with heterogeneous neurons. The aim of the study is to first of all investigate how the presence of diversity in the neuronal population affects sleep-wake transitions, since it is well-known that neurons are highly heterogeneous by their nature. In particular, within the orexin neurons population significant intrinsic diversity can be found: different electrophysiological properties, sizes in the diameter range 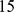
–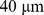, and various shapes such as a spherical, fusiform, or multipolar [19], [25], [22]. Secondly, also stochastic fluctuations, representing current noise, are added to the model and the response of the system is studied for different levels of noise. The question naturally arises, to what extent noise and diversity are essential ingredients for the functioning of assemblies of neurons and other complex systems, and what is the optimal level of noise and diversity required for the emergence of an optimal response to external stimuli. It is shown below that the model under study presents both diversity-induced resonance and stochastic resonance, but the former appears more clear and robust, since it is always associated with a regular almost-periodic spiking-silence activity, rather than to the irregular random transitions characterizing the stochastic resonance regime.

## Materials and Methods

In this section the two-neuron model of sleep-wake cycles [18] is described and some examples of dynamics in the presence of an external periodic signal are illustrated. Further, this model is extended to account for multiple neurons dynamics and heterogeneity, and a simple quantitative criterion to estimate the quality of a sleep-wake cycle is introduced. This criterion will be used in the Results section to compare sleep-wake cycles dynamics obtained at different parameter sets.

### The two-neuron model

**Figure 1:**
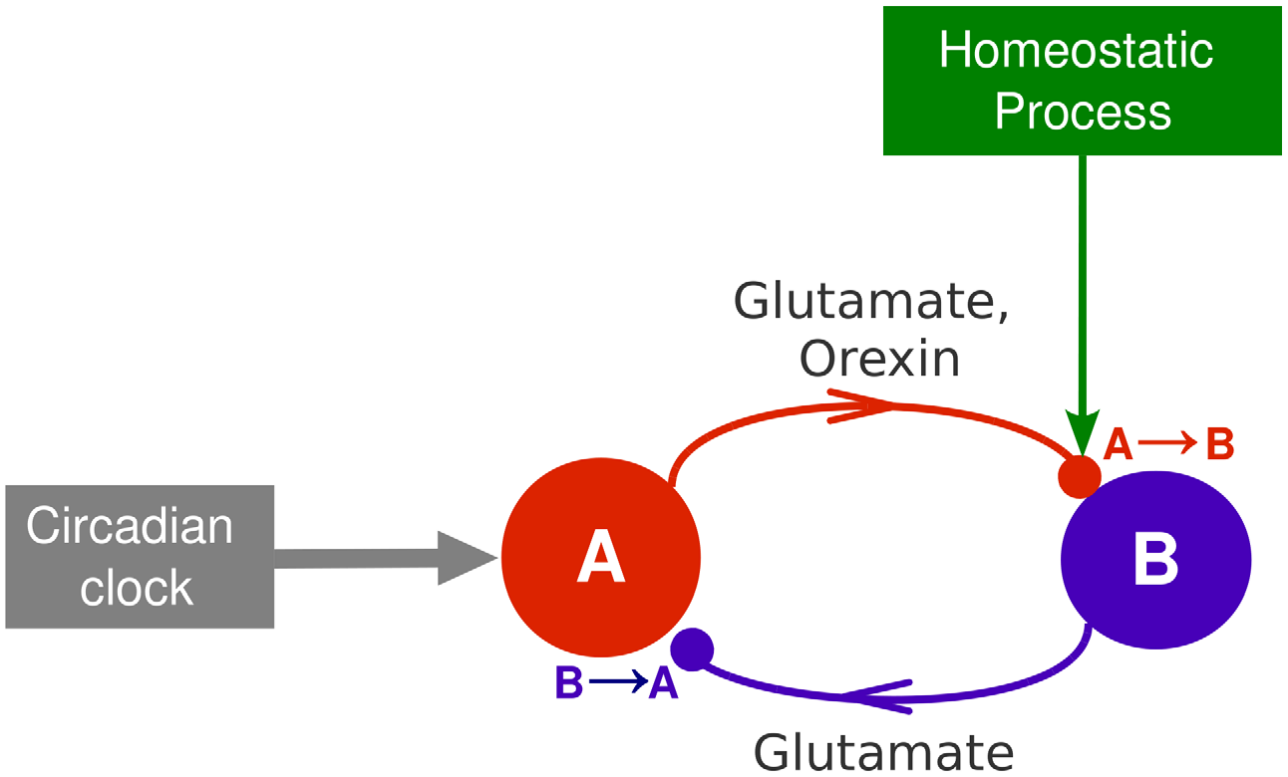
Scheme of the two-neuron model of the sleep-wake cycle [18]. The 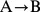 red arrow from the orexin-producing neuron A (red circle) to the neuron B (blue circle) represents the glutamate projection as well as the orexin projection regulating the homeostatic process. The blue arrow represents the 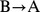 glutamate projection. The neuron A is also acted upon by a periodic signal representing the effect of the circadian clock.

The original model of the homeostatic regulation of sleep has a minimal structure consisting of two representative interacting neurons A and B, as depicted in Fig. 1. The neuron A simulates a representative neuron from the orexinergic neuronal population, while the neuron B represents a local glutamate interneuron (for details see [18]). The state of wakefulness or sleep is determined by the firing regime of neurons A and B, since these neurons are known to fire during wakefulness and be almost silent during sleep (see e.g. [22]).

Interaction between the neurons A and B takes place through glutamate and orexin neurotransmitters, as detailed below. The neuron A is acted upon by a stimulus in pace with the circadian rhythm, here treated as a periodic external signal — a simplification justified by its independence from the homeostatic process [26]. The homeostatic process itself is described by an additional macroscopic variable 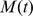 simulating availability of orexin.

Dynamics of the neurons A and B are based on a Hodgkin-Huxley-type model [27]. The membrane potentials of the neurons A (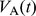) and B (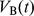) are thus calculated as:
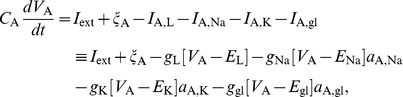
(1)

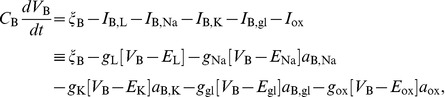
(2) where 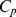 (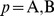) are the membrane capacitances per unit area of the respective neurons, 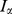 (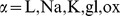) are the ionic currents, 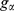 are the maximum conductances, and 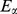 are the equilibrium potentials. The capacitance values are taken as 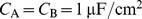. The values of all the other model parameters are listed in Table 1.

**Table 1:** Parameters of the two-neuron model [18].

	Conductance ( 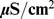 )	Equilibrium Potential ( 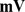 )	Slope Parameter ( 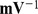 )	Threshold Potential ( 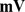 )	Time Scales ( 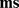 )
L (Leakage current)	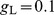	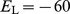			
Na (Sodium current)	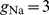	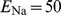	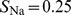	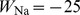	( 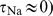
K (Potassium current)	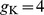	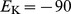	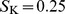	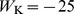	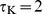
gl (Glutamate current)	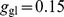	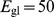	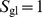	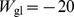	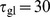
ox (Orexin current)	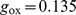	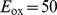	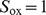	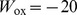	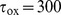
	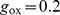				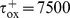
					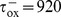
Periodic current					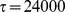
					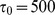

In the following we give a detailed explanation of different parts of the model.

• *External forces*. The current 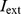 acting on the neuron A and the noise currents 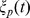, 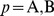, can be considered as external forces, in the sense that they do not depend on the system variables.

**Figure 2:**
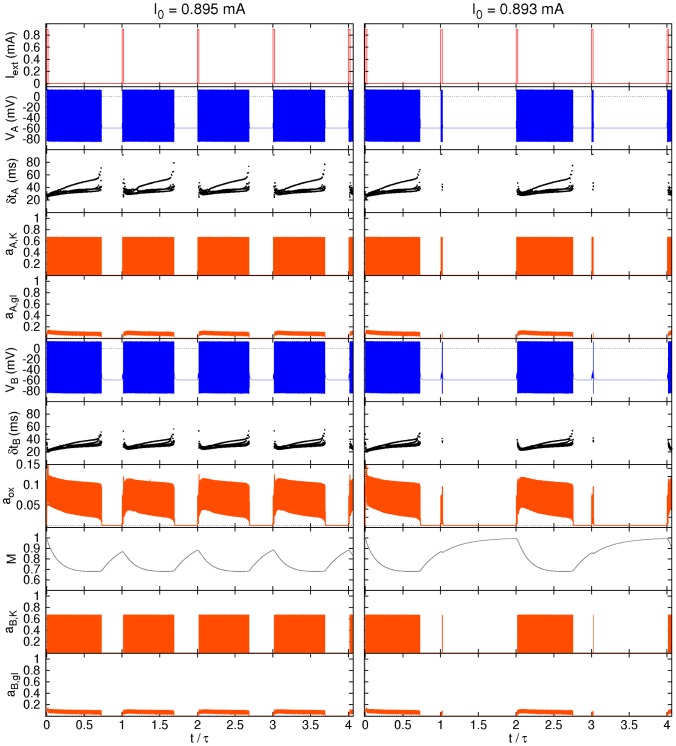
Response of the two-neuron model. Main variables and inter-spike times 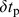 (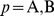) *versus* time for a pulse height 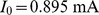 (left) and 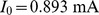 (right), see text for details.

The *external current* 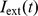 is assumed to simulate a stimulus associated with the circadian rhythm. For simplicity in the present study a periodic pulse input is used to introduce circadian activation of the system: 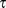, 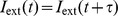. Such current can be interpreted as an awakening effect of an alarm clock or some other disturbance coming with a period of 24 hours. In the following we employ a train of rectangular pulses with length 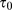 (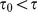) and height 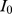, as depicted in Fig. 2-top,
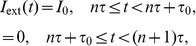
(3) where 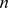 is an integer. This simple form is chosen because it is convenient for carrying out a systematic study of the neuron response at different parameters sets. However, it should be kept in mind that it represents a drastic simplification, and more realistic shapes of circadian currents can also be used [18].

The *noise* term 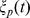 represents fluctuating currents that are known to be always present in neurons. For simplicity, we assume zero-average Gaussian white-noise processes:
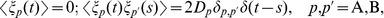
(4) with 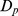 being the noise intensity.

• *Internal dynamics*. The leakage, sodium, and potassium currents 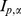 (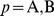; 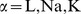) in the equation of the neuron 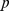 depend only on the variables of the *same* neuron 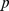 and, thus, describe the neuronal internal dynamics.

The *leakage currents* 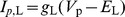 represent a flow of ions with a small conductance 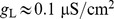 driving the membrane potential toward the negative value 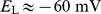.

The depolarizing *Na-currents* 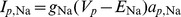 have a maximum conductance 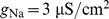 and a large positive equilibrium potential 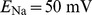. The activation variables 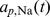, with 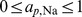, represent the fraction of open ion-channels contributing to the Na current. Because of their fast activation relative to the other time scales, the Na-current is assumed to be activated instantaneously, according to its voltage-dependency:
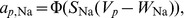
(5) where 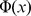 is the sigmoid function
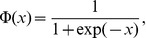
(6)

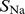
 is the steepness of the sigmoid function and 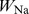 is the half-activation potential.

The repolarizing *K-currents* 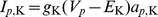 are characterized by a maximum conductance 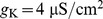, a large negative equilibrium potential 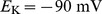, and a longer activation time than the depolarizing Na-current, namely 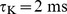. Consequently, the dynamics of the K-currents activation variables are modelled as
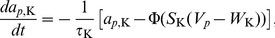
(7) where 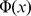 is defined in Eq. (6).

*Couplings*. The neurons A and B are mutually coupled by chemical synapses through the glutamate-induced (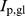) and the orexin-induced (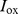) currents. Unlike the Na and K currents, 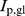 and 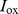 depend on the activity of both presynaptic and postsynaptic neurons. The activation variables 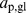 and 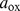 depend on the appearance of a spike in the presynaptic neuron, i.e. on the presynaptic voltage. Additionally these currents depend on the voltage of the postsynaptic neuron, similarly to other ionic currents. Both glutamate and orexin are excitatory neurotransmitters, so they are assumed to open depolarizing ion channels, such as Na-channels.

The activations of the glutamate-induced currents are modeled as:
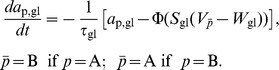
(8) This equation is similar to Eq. (7) but has the important difference that the equilibrium value 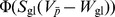 for the activation variable 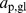 depends on the membrane potential 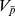 of the *other* neuron 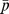 (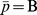 if 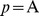, 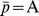 if 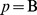). The time constant 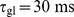 accounts for the delay coming from the activation of glutamate receptors, and the following activation of ion channels.

The *orexin-induced current* represents the effect of orexin produced by the neuron A and acting on the neuron B. It is modeled in a form similar to the glutamate-induced current. This current provides a simplified description of the effects of orexin on the neuron B which appear after a complex series of processes, involving production of orexin in the soma of the neurons, its release in the synaptic cleft, and activation of G-protein coupled metabotropic receptors. The dynamics of the activation variable 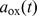 depend not only on the membrane potential 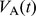, but are also related to the availability of orexin at time 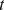, described by the additional variable 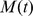 (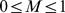). The dynamics of the variables 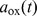 and 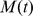 are defined by the equations:
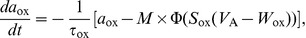
(9)

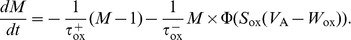
(10) The term 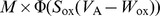 in the Eq. (9) reflects activation of the synaptic current due to appearance of a spike in the presynaptic neuron A. At the same time it determines the rate of orexin availability reduction in Eq. (10) due to spiking of the neuron A with a time constant 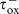. The first term in Eq. (10) determines recovery rate of the orexin availability with time constant 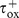.

The meaning of the product 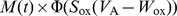 is that there is orexin-induced activity in neuron B if (1) there is enough orexin available above a critical threshold [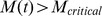], and (2) the neuron A is in the firing state [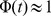].

The time constants 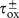 accounting for the orexin dynamics are much longer than the time constants associated with ionic current terms. The time constant 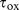 of the homeostatic regulation process is even longer, being of the order of magnitude of the daily period 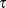.

For numerical convenience, simulations are made over rescaled daily and orexin time scales: the daily period was assumed to be 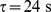, instead of 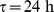, achieved through a suitable rescaling, which was applied to the orexin time scale 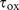 and the production and reduction times 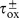. The other time parameters are left unchanged. Since such rescaled 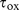 and 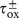 are still much larger than any other time scale of the microscopic dynamics, the rescaling does not change the main results of the simulations. See [18] for a detailed validation of such rescaling procedure.

All the parameter values for the currents are listed in Table 1. It is assumed that the neurons A and B share the same parameter values, unless specified otherwise. Such an assumption is justified, because the major properties of these neurons required for the model are the tonic firing (periodic single spike activity) and silent states. Without any external input both neurons should be in a silent state, while they are brought to firing activity in response to depolarization. Therefore, change of parameters in a physiologically allowed range would primarily lead to the different amount of depolarization needed to excite neurons, and would not affect the major outcomes of the simulations.

The system defined above is essentially an excitable feedback system, i.e. both the external input of sufficient strength and the AB coupling are essential elements for maintaining firing activity of the neurons. Orexin-related dynamics, with the associated long time scales, are expected to direct the homeostatic sleep process, which regulates the sleep-wake transitions. The healthy sleep-wake cycles in this system are realized as follows:

- *Initiating wakefulness*. A sufficiently strong or long external signal or a stimulus associated to the circadian rhythm, e.g. the idealized rectangular pulse considered here, activates the system and induces firing activity in the neuron A. Due to the excitatory synaptic connection from A to B, the neuron B is also activated.
- *Maintaining wakefulness*. Once the pulse is finished and the external current is zero, the system remains in the wake state (i.e. both neurons A and B are firing) due to reciprocal excitation between the neurons. The firing activity lasts for a fraction 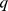 of the daily period 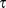. Ideally one can assume 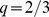, corresponding to 16 hours for a day of 24 hours, i.e. 16 seconds for the daily period 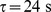 with the time scales of the model considered here.
- *Initiating sleep*. The firing stops in both neurons due to decreased availability of orexin according to the dynamics of 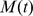. This is associated witch the transition from wake to sleep.

Two examples of the two-neuron model dynamics without noise are illustrated in Fig. 2. The left part of the figure represents the response obtained for a pulse length 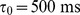 and height 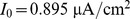. In each period orexin is depleted during the neuronal activity and recovered while the neurons are silent. The stimulus parameters used in this example have been intentionally chosen close to the critical firing threshold, so that by slightly reducing the pulse height or length, the periodic appearance of a continuous time interval of spiking regime is lost. Such case is demonstrated in the right hand side of the figure, where the current pulse height is slightly lower, 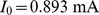, while all the other parameters are kept the same. There the prolonged wake state is induced only every other day, because the input is insufficient to induce sustained spiking at the same levels of orexin availability. By reducing the pulse amplitude or duration even further it is possible to observe different behaviors such as triple or higher-order periodicities.

### The heterogeneous model

As a step toward a more realistic model we generalize the two-neuron model into a heterogeneous multi-neuron model. For simplicity we first increase the number of orexin neurons only. To do this we replace the single neuron A by a set of 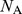 neurons 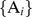 (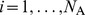), while still maintaining only one neuron B. Also, in this paper we assume that the diversity is constant in time in order to consider the simplest case possible.

In reality a certain level of heterogeneity is observed in all neuronal parameters. However, given that our model neurons are simple pacemaking neurons such diversification of different model parameters (in a physiologically allowed range) would simply lead to slightly different firing rates of the neurons. This, in turn, will result in diversity in activations of synaptic currents, which can be mimicked by simply diversifying their activation thresholds. Thus, in the following we can limit ourselves to studying the effects of diversity in activation thresholds of synaptic currents without loss of generality. Furthermore, as a first step, the heterogeneity is only introduced in the glutamate-induced currents to avoid having a too complicated system, which would become difficult to understand.

With regard to the coupling topology among the orexin neurons, so far there is no detailed experimental data. Therefore, for simplicity, we chose an all-to-all coupling via gap junctions, but other variations can be tested in the future. The intensity of the coupling has been chosen large enough to ensure that the neurons 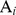 respond in pace to the external current. The equations of the two-neuron model are modified accordingly.

• *Dynamics of the neurons* 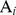. The membrane potentials 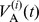 of the neurons 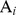, are described by equations analogous to Eq. (1):
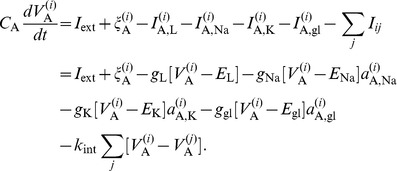
(11) The current terms are similar to those in the two-neuron model, apart from the additional coupling currents between two generic neurons 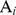 and 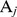, 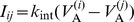, with 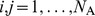, where 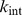 is the gap junctions conductance that can be treated as coupling strength. The currents' activation variables 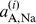 and 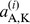 are modeled in accord with the equations of the two-neuron model. Note that the specific values of the activation variables will be different for different neurons since they depend on voltages of each particular neuron 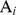.

**Figure 3:**
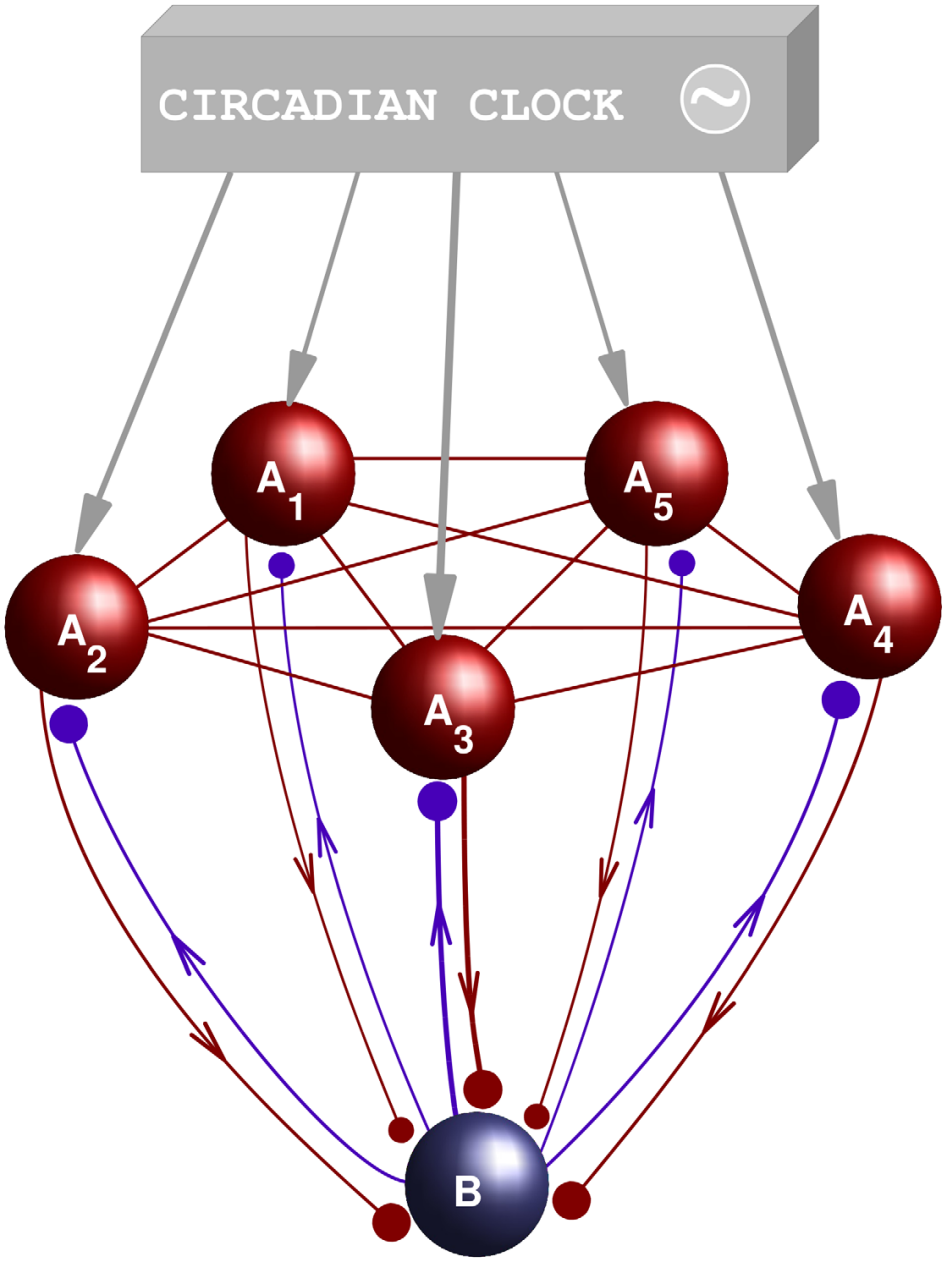
Scheme of the heterogeneous model. Example of model system with 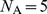 orexin-producing neurons 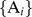, 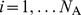 (red spheres) and one neuron B (blue sphere). The neurons A interact with each other through an all-to-all coupling (red lines). Blue and red projections have a meaning similar to those of Fig. 1: the neuron B is coupled to the neurons 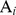 through parallel glutamate projections, while each neuron 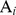 is coupled to neuron B through a glutamate and an orexin projection. The neurons A are also acted upon by a stimulus representing the effect of the circadian clock (gray arrows).

For simplicity the same external current 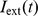 given by Eq. (3) is assumed to act on all neurons 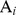 (see Fig. 3). The noise terms 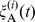 as well as the noise 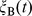 acting on the neuron B (see below) are also defined similarly and assumed to be statistically independent from each other. For convenience the properties of all stochastic forces are written together (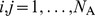):
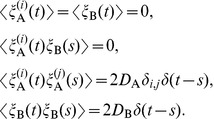
(12)


• *Connections from the neuron B to the neurons* 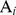. The neuron B has glutamatergic synaptic inputs to each of the neurons 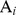 as depicted in Fig. 3. Diversity is introduced in the activation thresholds of the glutamate-induced currents according to the following equation for the activation variables:
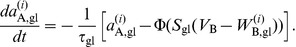
(13) The thresholds 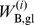 adopt different values for each neuron 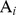 that are independently extracted from a probability distribution defined later in the text.

• *Connections from the neurons* 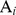 *to the neuron B*. Each of the neurons 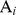 has synaptic projections to the neuron B. This is translated in the model by replacing the single glutamate- and orexin-induced currents with their averages such that Eqs. (8) and (9) for the activation variables become:
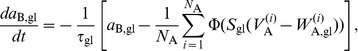
(14)

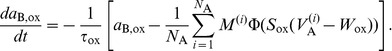
(15)


Note that diversity is again introduced in the activation thresholds of the glutamate-induced currents 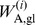 corresponding to heterogeneous 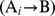 synapses located at the neuron B. Due to the differences in the 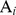 neurons, the orexin availability function 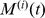 is different for different neurons, although still following Eq. (10).

The above described set of equations constitutes the multi-neuron heterogeneous model of the homeostatic regulation of sleep. Numerical results were obtained using a variation of the Runge-Kutta 2nd-order method, which is suitable for equations with stochastic terms, namely the Heun method [28]. Identical initial conditions were assumed for all neurons, corresponding to a silent state.

### Quantifying the quality of the sleep-wake cycle

In this section a heuristic criterion is introduced in order to evaluate and compare the quality of the system responses obtained for different external signals or internal parameter values.

For this purpose, the period 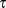 is divided into a “day” wakefulness sub-period of length 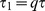 and a “night” sleep sub-period of length 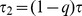, with 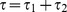. The quantity 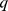 is defined as a *wake fraction*. A typical sleep-wake cycle with an eight-hour sleep sub-period has 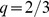. For the day corresponding to the 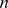-th period 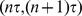, the “day” is represented by the sub-interval 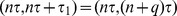, which covers the first fraction 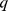 of the period, while the “night” extends in the complementary fraction 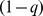 of the period in the time interval 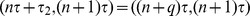.

For each period 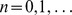, we compute wakefulness time intervals 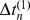 and 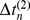 spent by the system in the wake state during the day, 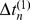, and night, 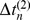. The wake/sleep state is identified with the spiking/silent regime. A simple quantitative estimate of the quality of the sleep-wake cycle can, thus, be done through the following linear function of the wakefulness time intervals,
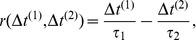
(16) where 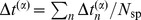, 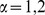, represent the average of the wakefulness time intervals during the day (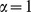) and during the night (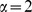), with 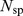 being the total number of periods of the simulation.

The fractions 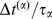 (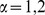) can vary in the interval 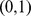; then the coefficient 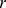 in Eq. (16) is limited in the interval 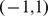. The maximum value 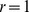 corresponds to an optimal cycle with 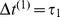 (wakefulness during the entire day) and 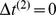 (sleep during the entire night); any deviation from the optimal state (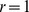) comes either from values 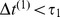 (implying some sleep during the day) or values 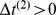 (meaning at least some wakefulness in the night). See Text S1 for further details on the definition of the time intervals 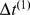, 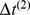 and the coefficient 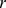.

## Results

In this section we study how the presence of disorder affects the system response and discuss the main differences compared to the two-neuron model. The term “disorder” is used here to refer to either *noise*, i.e. disorder in time (stochastic terms in the external current), or *diversity*, i.e a quenched heterogeneity in the neuronal parameters. These two aspects are studied separately. For the sake of simplicity, we examine the response of the system to a periodic stimulus represented by a train of short rectangular pulses as defined in Materials and Methods.

In each of the examples considered, the initial configuration in the absence of noise and diversity is the same as the sub-threshold state illustrated in Fig. 2-right with a double-periodic response. It is obtained for a reduced height of the current pulse 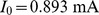, while the other parameters are unchanged as given in Table 1. The reason for starting from such an under-threshold non-optimal configuration is that it is most sensitive and, thus, best illustrates the effects of added noise or heterogeneity. While a response with a double-periodicity may seem unrealistic, this starting configuration is intended to be an example of non-optimal response rather than a standard reference state. In fact, in realistic situations noise and heterogeneity are always present so that such a state without noise or diversity represents a hypothetical system that would be obtained if one could switch off noise or replace heterogeneous synapses with perfectly identical ones. The results presented below suggest that a multi-periodic sleep-wake cycle can be turned into a regular (single-periodic) one by adding a suitable degree of disorder.

### Effect of noise

Here we investigate the effects of the noise currents in the equations for the membrane potentials. For clarity only the cases in which noise currents are present either in the neurons 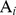 or in the neuron B are considered.

#### Noise in the neurons 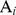


**Figure 4:**
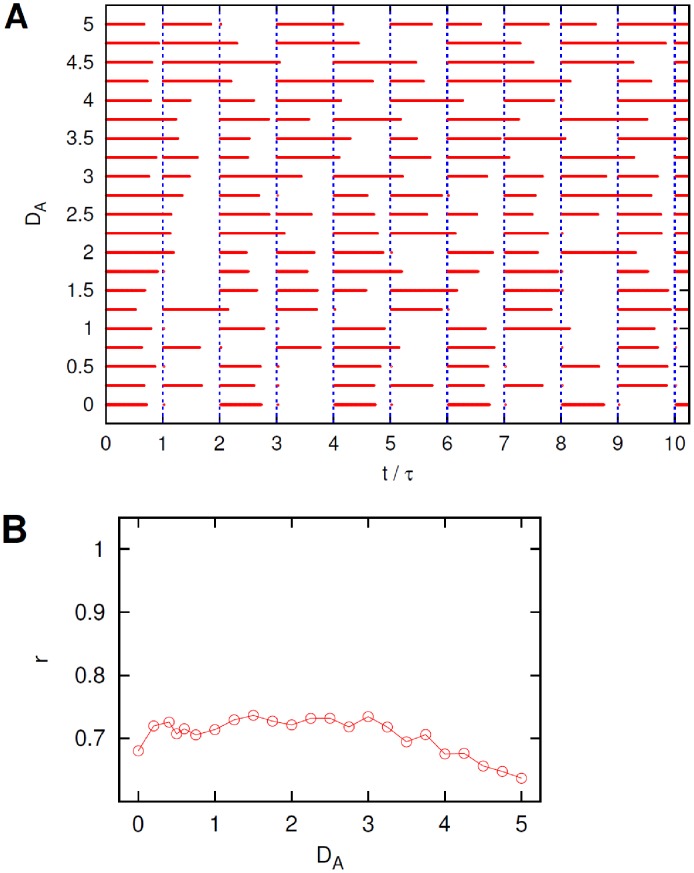
Effect of noise in neurons 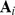. (A). Ten periods of the raster plots of neuron B for different intensities 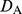 of the noise acting on neurons 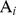. Vertical dashed lines mark the beginning of the pulses of the external current 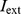, see text for details. (B). Coefficient 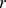, from Eq. (16), *versus* current noise intensity 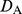.

To study the effects of the noise currents 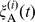, 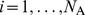 acting only on the neurons 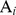 (as per Eq. (11)) we set 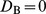. Also, no diversity in the characteristic parameters of neurons 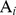 is introduced. We have simulated a system with 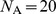 identical neurons and a single B neuron on a time interval 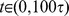. A raster plot for the activity of the neuron B at different values of 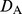 (indicated on the left) is shown in Fig. 4-A. The plot shows that

- for small 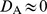 the system's configuration corresponds to the assumed non-optimal double-periodic solution;
- the system's response becomes slightly more regular and periodic as 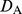 is increased, despite the fact that the neuron cannot initiate a firing event at the beginning of each period;
- as 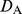 becomes even larger the neuron B keeps firing tonically for a longer and longer time interval (even longer than a single period) thus deteriorating the general quality of the response.

**Figure 5:**
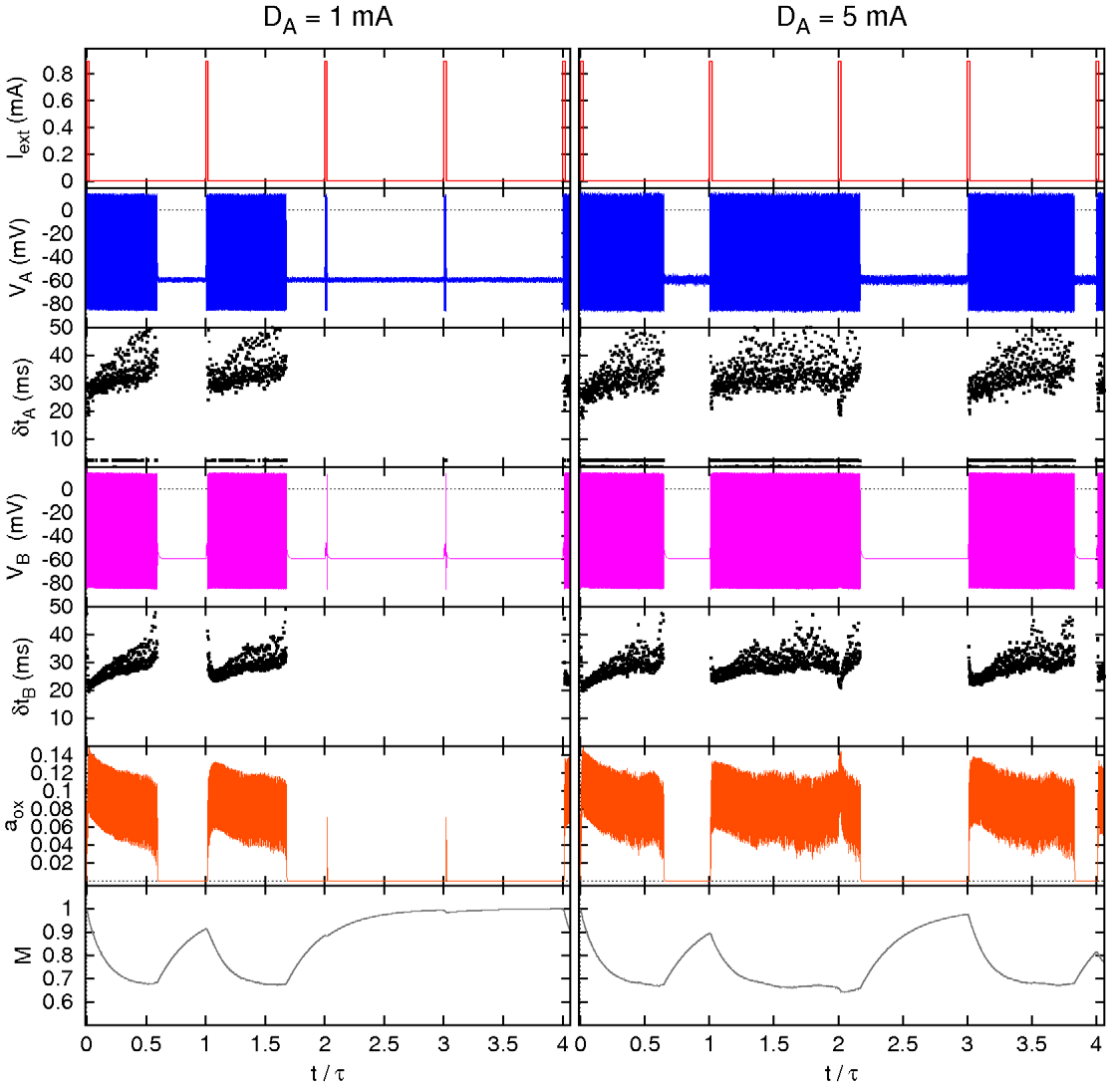
Effect of noise in neurons A. Sample of four periods of some relevant variables and inter-spike times 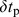 (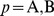) *versus* time of neuron 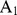 and neuron B for an intensity of noise in neurons A 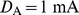 (left) and 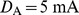 (right). Compare Fig. 4 and see text for details.

A sample of time dependence of the main variables in the interval 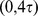 for 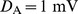 and 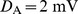 is illustrated in Fig. 5. In general, the type of variability induced by noise currents acting on the neurons 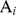 affects both the firing initiation and, especially, its duration. However, it is difficult to establish an actual improvement of the quality of such a response as a function of the noise intensity 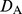, as even the coefficient 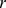, shown in Fig. 4-B, suggests only a mild stochastic resonant behavior characterized by a wide plateau at intermediate values of 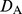.

#### Noise in the neuron B

**Figure 6:**
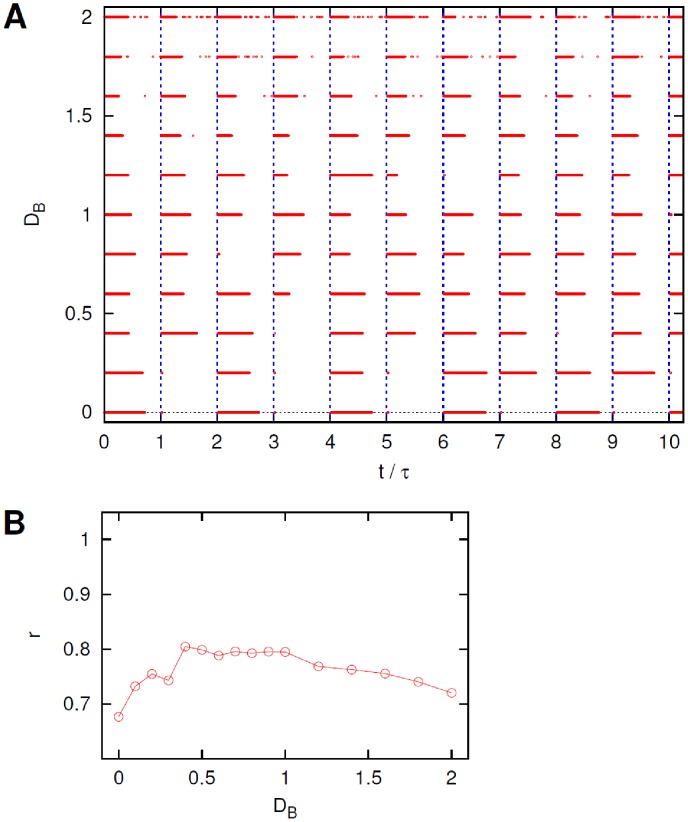
Effect of noise in neuron B. (A). Sample of ten periods of the raster plots of neuron B for different values of the intensity 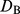 of the noise acting on neuron B. Vertical dashed lines mark the beginning of the pulses of the external current 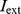, see text for details. (B). Quality of the sleep-wake cycle from the coefficient 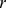, Eq. (16), *versus* current noise intensity 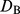.

Here we consider the complementary case, in which 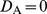 and a current noise only affects the neuron B. A sample of raster plots of the membrane potential of the neuron B is depicted in Fig. 6-A in the time window 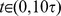 for the values of the noise intensity 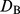 indicated on the vertical axis.

The raster plot in Fig. 6-A indicates that:

- the smallest values of noise intensity 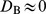 correspond to the double-periodic configuration discussed above;
- the response becomes periodic, and the length of the firing periods more regular for higher values of 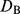;
- at larger values of 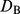 the state of sleep is frequently interrupted by almost isolated spikes at random times.

**Figure 7:**
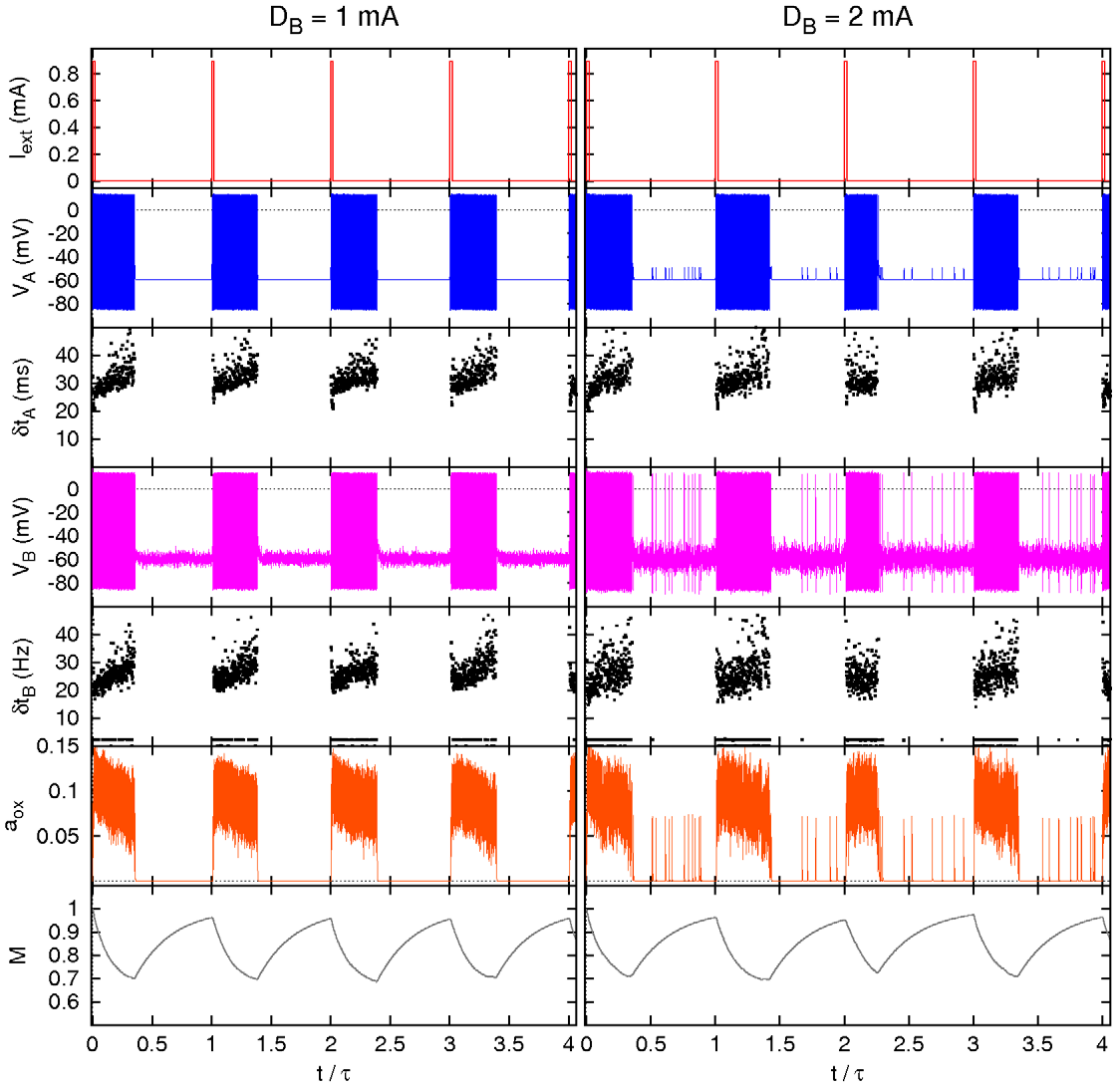
Effect of noise in neuron B. Sample of four periods for some relevant variables and inter-spike times 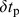 (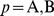) *versus* time for neuron 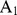 and neuron B for an intensity of the noise acting on neuron B 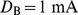 (left) and 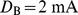 (right). Compare Fig. 6 and see text for details.

A representative example of time dependence of selected variables of the neurons 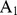 and B are shown in Fig. 7. Note the different type of behavior induced by a high levels of noise acting on the neuron B, compared to the case in which noise acts on the neurons 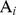. In the first case irregular switching between the firing and silent states is observed more often, especially considering the transient firings in the otherwise silent sleep state. Furthermore, this random firing appears only in the neuron B, but is insufficient to also induce spiking in the 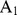 neuron. This activity may represents intermittent awakenings, which are likely due to the ability of noise to favor the ignition of spiking events. Such random spikes are not observed when noise acts on the neurons 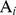 only, even at much larger noise intensities. This may be related to the coupling between the neurons 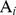, which constrains them in the same (spiking or silent) state. In order to excite all neurons 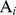 together one would need an input signal affecting all of them in the same way, which is highly improbable in a realistic system.

The dependence of the coefficient 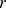 on 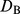 is shown in Fig. 6-B. Again, only a mild stochastic resonance behavior is suggested by the data when varying the noise intensity. It should be noted that in this particular configuration with noise acting only on the neuron B, the response of the neuron B does not depend on the number 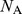 of homogeneous neurons 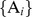, due to the equivalence to the configuration of the two-neuron model, as we have checked numerically. Thus, the plots of neuron 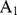 in Fig. 7 are representative of all other neurons 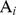. In fact, the external current 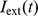 as well as the coupling currents are the same for each neuron 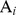, which produces the same response. According to the equations of the heterogeneous model, the effective current acting on the neuron B is the arithmetic average of the currents coming from the various neurons 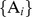 and, therefore, coincides with that of any single neuron 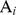. We use here a homogeneous multi-neuron generalized model only for a better comparison and consistency with the rest of this study.

### Effects of heterogeneity

The effects introduced by a heterogeneity in the neurons are dramatic compared to the effects of noise. The corresponding improvement of the system response for suitable intermediate amounts of diversity can be detected very clearly. This is the main result of this paper and it is illustrated in this section. Noiseless neurons are assumed for easier estimation of the heterogeneity effects (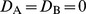).

As in the study of noise described above, we carry out the study of diversity starting from the same configuration with a non-optimal double-periodic response to the external periodic stimulus, corresponding to a zero diversity (homogeneous system). Heterogeneity is then introduced in the glutamate-induced currents, either in the thresholds 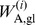 regulating the response of the 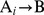 synapses at the neuron B or in the thresholds 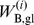 of the 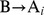 synapses at the neurons A. This is done by randomly extracting values 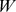 from a probability density 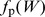 and assigning them to the threshold parameters 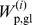 (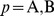). The probability density used here has a bell-shape 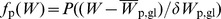, where 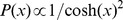, the quantity 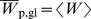 represents the average value, while 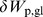 measures the dispersion of the distribution 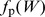 around the average value and is related to the standard deviation 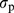 by 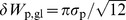. For further details see Text S1. The width 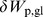 is assumed in the following as the measure of neuronal diversity. In order to carry out meaningful comparisons with the homogeneous (two-neuron) model, the average values are set equal to the corresponding parameters of the homogeneous two-neuron model,
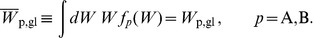
(17) The other parameters are unchanged compared to the two-neuron model, see Table 1.

#### Diversity in the 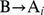 synapses (neurons 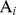)

Diversifying the potential thresholds 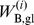 implies heterogeneous glutamate synapses located at the neurons 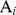, see Eq. (13) and Fig. 3. That is, each neuron 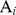 responds in a different way to the stimulation from the neuron B. Notice that this is a truly heterogeneous system which cannot be reduced to an effective two-neuron model—as in the case of heterogeneous synapses at neuron B considered in the next section. We studied a system with 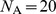 neurons 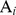 with diversified threshold parameters 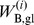, 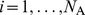. The system dynamics were examined for different sets of thresholds 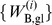 extracted from distributions 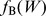 with different widths 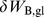 but always the same average value 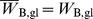.

**Figure 8:**
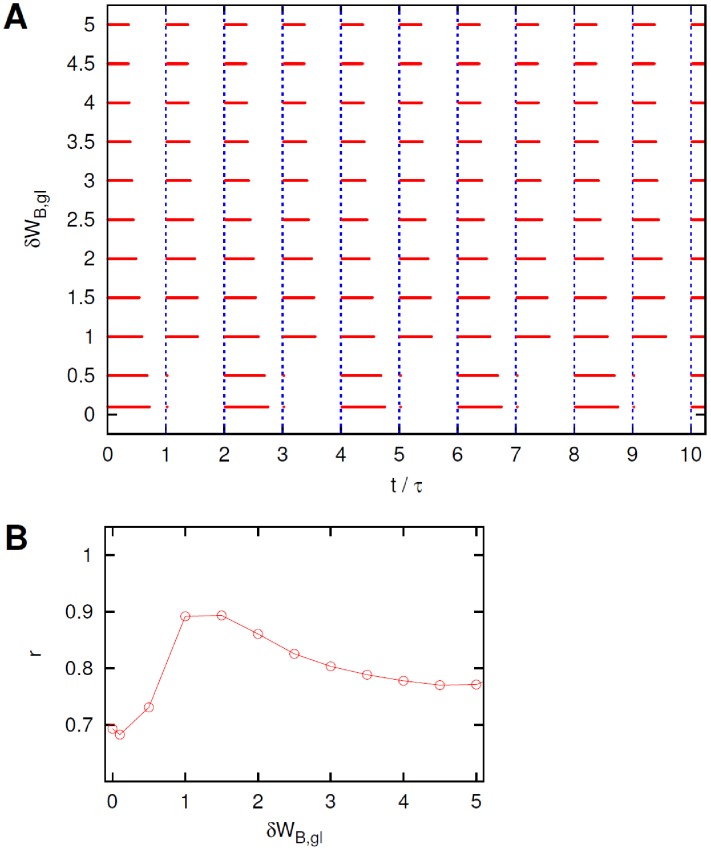
Effect of diversity in the 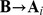 synapses. (A). Sample of ten periods of the raster plots of neuron B for different heterogeneity levels 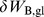 in the 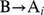 glutamate synapse thresholds, see text for details. (B). Quality of the sleep-wake cycle from the coefficient 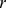, Eq. (16), for various threshold diversities 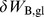.

**Figure 9:**
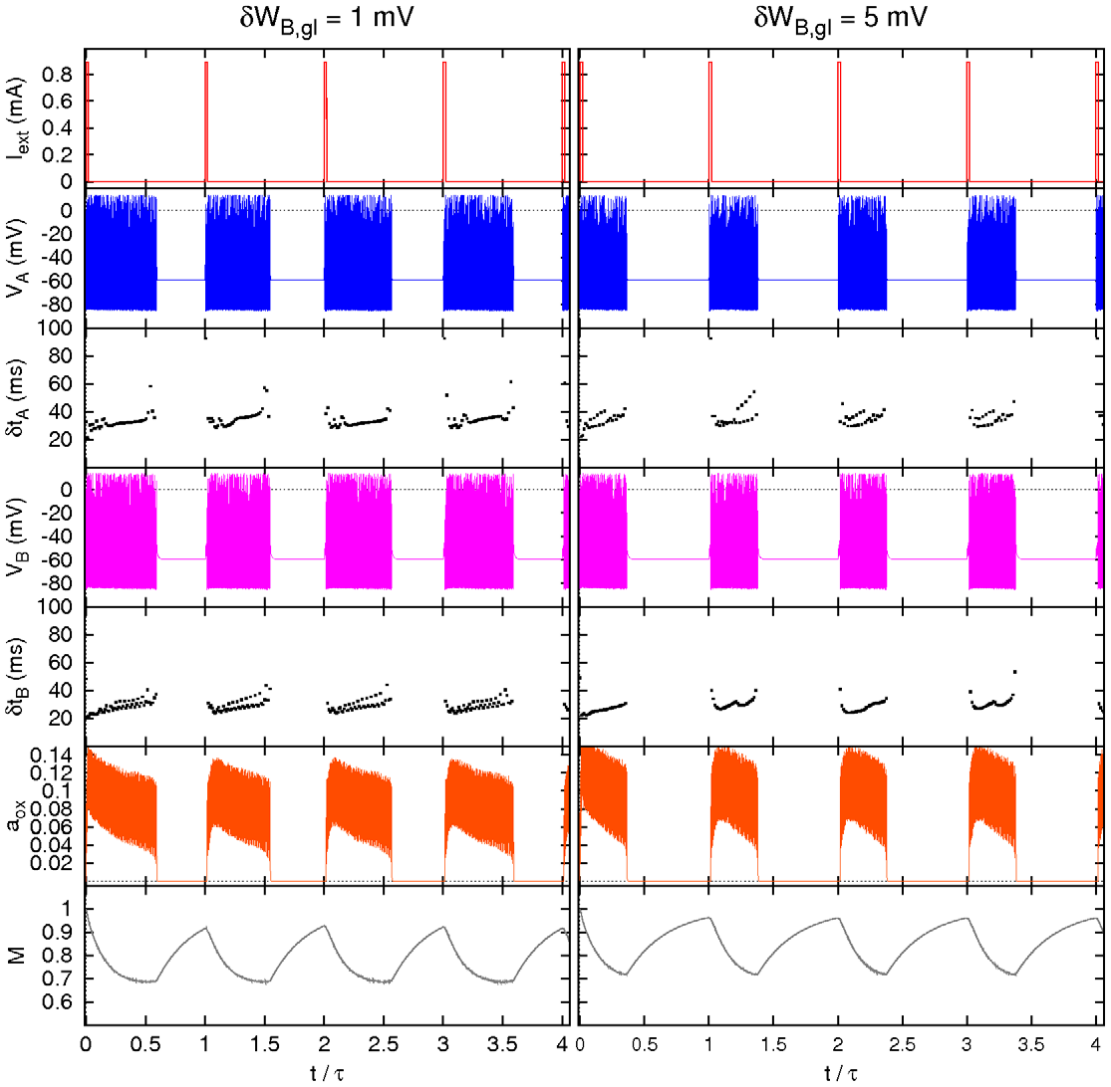
Effect of diversity in the 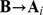 synapses. Sample of four periods of some relevant variables and inter-spike times 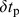 (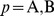) *versus* time for neuron 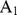 and neuron B for different levels of the threshold diversity 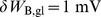 (left) and 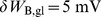 (right). Compare Fig. 8 and see text for details.

The resulting raster plots of the activity of the neuron B are shown in Fig. 8-A, and a sample of time dependencies for the neurons 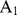 and B is shown in Fig. 9. The existence of an optimal degree of diversity, corresponding to a value 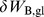 approximately between 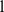 and 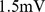, can be clearly seen both from Fig. 8-A and from the dependence of the coefficient 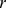 on the diversity degree 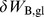, in Fig. 8-B.

The underlying mechanism leading the system from the double- to the single-periodic response as diversity is increased can be interpreted following the prototype mechanical model of diversity-induced resonance introduced in Ref. [3]. In this model a set of interacting oscillators moving in a bistable potential is subjected to an external periodic force, which pushes the system toward the left and the right barrier alternately. If the oscillators are identical, i.e. they have the same parameter values corresponding to an under-threshold regime, then the system of oscillators cannot perform jumps on the other site of the barrier under the action of the applied periodic force. However, when the parameters are diversified (keeping constant the corresponding average value) some oscillators respond more promptly to the force and jump to the other side of the barrier, gradually pulling the rest of the system. In the present case, each neuron 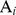 corresponds to a nonlinear oscillator of the example, while the parameter which is diversified is the activation thresholds 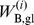 of the glutamate-induced currents.

**Figure 10:**
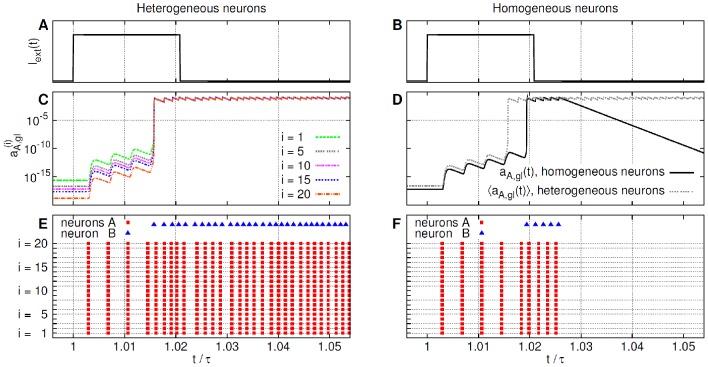
Effect of diversity in the 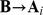 synapses. Comparison between the responses of the heterogeneous system (left column) and homogeneous system (right column) in the first part of the time period 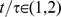 during the action of the 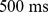 long current pulse starting at 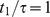 and ending at 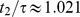. (A) and (B) (top panels). External current pulse. (C) and (D) (central panels). Behavior of some representative glutamate activation variables of the heterogeneous system, 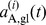 for 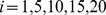 (panel (C)), and the (common) time dependence 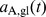 of the homogeneous system activation variables (panel (D), black continuous curve); in the latter figure also the average value 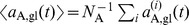 of the heterogeneous system (dashed grey curve) is shown for comparison. (E) and (F) (bottom panels). Raster plots of all the neurons of the system. See text for further details.

To show that this is the actual mechanism in action, Fig. 10 (left) illustrates the response of the heterogeneous system by depicting the time dependence of the glutamate activation variables 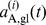 of the neurons 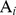, 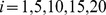, with different values of the thresholds 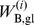, at the beginning of a new period in the presence of the periodic current pulse. In Fig. 10 also the raster plots for all neurons in the same time interval are shown. One can notice that the activation variables 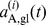 behave differently from each other. Those associated to the lowest values of the activation threshold (indicated by small 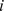 values) respond stronger to the current pulse than those with the highest values of the threshold (largest values of 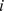). The system is observed to reach the spiking regime faster than in the homogeneous case, which is shown in the right part of Fig. 10 through the comparison between the glutamate activation variable of the homogeneous system, 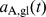, and the average activation variable 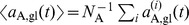 of the heterogeneous system. Eventually, 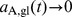 and the homogeneous system goes back to the silent state, while the average activation variables of the heterogeneous system (and their average 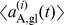) continue to oscillate around positive values, signaling the stability of the reached firing state.

#### Diversity in the 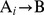 synapses (neuron B)

In order to study the effects of added heterogeneity in the glutamate synapses located at the neuron B, one has to diversify the potential threshold parameters 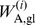, see Eq. (14). For this particular case, it is possible to simplify the model into a two-neuron model with a single effective AB coupling. This is possible because heterogeneity only enters Eq. (14), while other model equations reduce to the same equations in the case of identical neurons 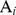, so that all neurons 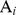 behave in the same way. Thus, the effective glutamate-induced current to the neuron B is
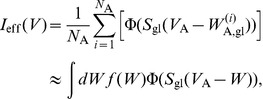
(18) where 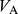 is the common value of the membrane potentials of the neurons 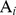. Here 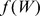 is the probability density of the corresponding thresholds 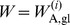, which is assumed to be the same bell-shaped probability density 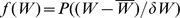 as discussed above, with 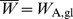 and 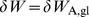.

We now consider two limiting cases of the effective current given by Eq. (18). In the limit 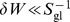, when diversity is very small on the scale 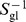, it can be assumed that the following approximation holds, 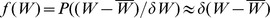 and the integral (18) can be reduced to the homogeneous result,
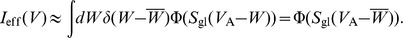
(19) In the complementary limit of high diversity level, 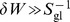, the smooth function 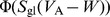 can be approximated with Heaviside step functions 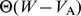, and the effective current becomes
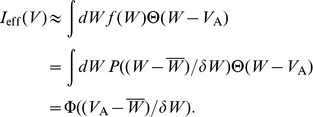
(20) This follows from the form of the chosen distribution, 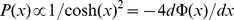. Thus, in both these limiting cases the effective current can be written in the form 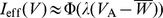, with 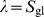 for 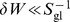 and 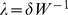 for 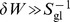. It is interesting that, as we have checked by numerical integration of Eq. (18), the same analytical form also holds for intermediate values of 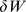 and 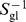, so to a very good approximation the effective current can be written as 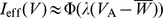, where the parameter 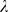 depends on the ratio 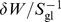 and varies monotonously between 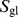 and 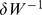, as 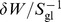 varies between 0 and 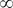.

**Figure 11:**
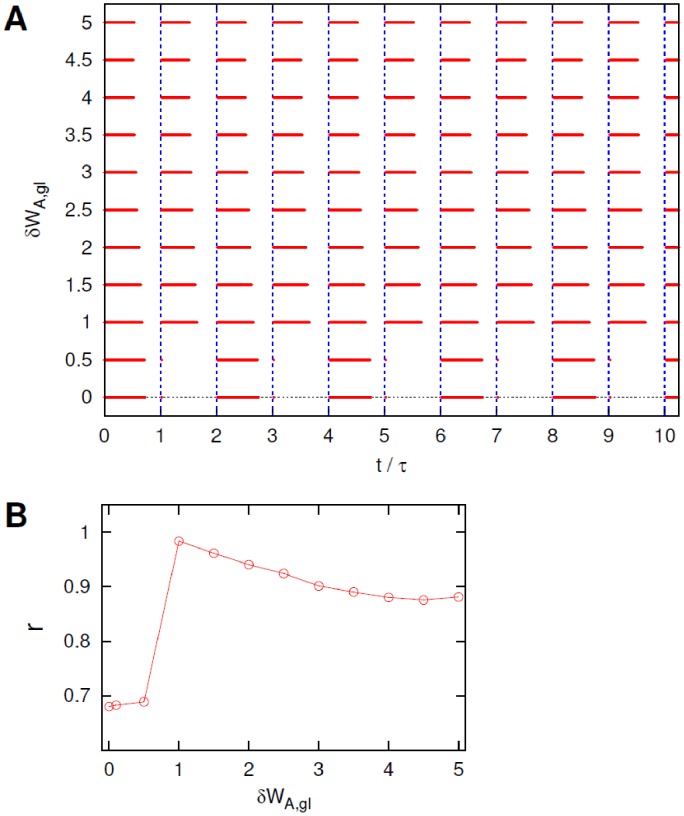
Effect of diversity in the 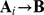 synapses. (A). Sample of ten periods of raster plots of neuron B for different heterogeneity levels 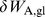 in the 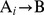 glutamate synapse thresholds, see text for details. (B). Coefficient 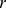, Eq. (16), for various degrees of diversity 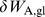.

**Figure 12:**
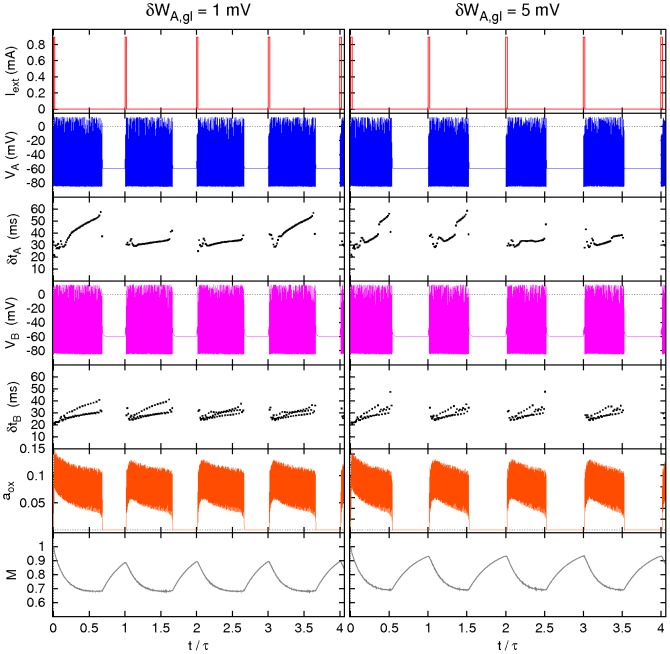
Effect of diversity in the 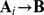 synapses. Sample of four periods of the time dependence of some relevant system variables and inter-spike times 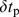 (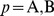) of neuron 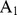 and neuron B for a threshold diversity 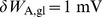 (left) and 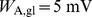 (right). Compare Fig. 11 and see text for details.

The system's response at different levels of heterogeneity, 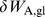, is presented in Fig. 11-A through the raster plots for the neuron B. A sample of time dependence is shown in Fig. 12, while Fig. 11-B shows the dependence of the coefficient 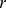 on the diversity level. In Fig. 11, it can be seen that for small values of the diversity 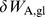 the response of the neuron B presents the double periodicity of the reference configuration. Single periodicity is recovered for higher levels of diversity. At even higher values of 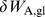, the coefficient 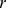 begins to decrease. The points of the curve corresponding to the highest values of 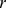 suggest an optimal degree of diversity 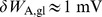.

The main difference compared to the case in which noise intensity is varied, is that the response of the system remains more regular also at the highest levels of diversity considered, i.e. without random spikes appearing during the silent state and with a typical cycle well shared between a day and a night sub-period.

## Discussion

In the present work we have introduced a heterogeneous multi-neuron version of the previously developed physiologically motivated model of the homeostatic regulation of sleep. The multi-neuron model is composed of a population of conductance-based orexin-producing neurons and a single representative glutamatergic neuron. In this model the glutamatergic and orexinergic neurons are undergoing transitions between firing and silence depending on the external circadian input and internal homeostatic mechanisms. These transitions correspond to the transitions between wake (firing) and sleep (silence), with the homeostatic mechanism being dependent on the availability of orexin.

The specific aim of this study was to explore the effects of noise and diversity in the regulation of sleep-wake cycles in such a model. It is clear that diversity and noise are integral parts of all biological systems, including the orexinergic neuronal population in the lateral hypothalamus. However, the role of disorder, and especially diversity, is rarely considered in the physiologically based mathematical models of sleep-related systems [29], [30], [31], [32], [33], [34], [35]. To our knowledge, diversity had so far been included only in one such model, i.e. the model of interacting circadian oscillators [5], and here we present another example of the constructive role of diversity in regulation of sleep.

We have demonstrated the existence of a diversity-induced resonance, leading to a clear and strong improvement of the quality of the sleep-wake cycles, at a physiologically justified intermediate level of diversity of the orexin-producing neurons. However, only a mild improvement was found with varying noise intensity (stochastic resonance phenomena).

We have considered the simplest system with only 20 heterogeneous orexin neurons and one local glutamate neuron. Also we have used a very simple all-to-all network topology for the connections among orexinergic neurons. However, it can be expected that constructive effects of diversity will be found also in other model configurations. In the future, more realistic modifications of the model with a larger population of glutamatergic neurons and more sophisticated inter-populations connections should be considered. Furthermore, in the future studies interplay between noise and diversity should likewise be investigated, since in nature both types of disorder are normally present.

The validity of the result obtained within this model may be more general, since diversity-induced resonance is known to take place for suitable values of the parameters in general networks of interacting (non-linear) oscillators. A question then naturally arises: whether the phenomena encountered here could also characterize other systems where there is a coupling between two very different time scales or, in other words, if homeostatically regulated biological systems may take advantage from a suitable level of heterogeneity of their components.

## Supporting Information

Text S1
**Some details on the numerical simulation.** The supporting Information file Text S1 contains some more detailed information about: A: the definition of the wakefulness time intervals during the day and the night, 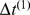 and 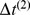, respectively, used for evaluating the quality of the sleep-wake cycle; B: the extraction procedure of the diversified threshold potentials 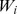 and the form of the corresponding probability distribution 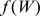.(PDF)

## References

[1] von Economo C (2009) Cellular Structure of the Human Cerebral Cortex. Basel: S. Karger AG.

[2] Gerashchenko D, Shiromani PJ (2004) Different neuronal phenotypes in the lateral hypothalamus and their Role in sleep and wakefulness. Mol Neurobiol 29: 41–59. 15034222 10.1385/MN:29:1:41

[3] Tessone C, Mirasso C, Toral R, Gunton J (2006) Diversity-induced resonance. Phys Rev Lett 97: 194101. 17155633 10.1103/PhysRevLett.97.194101

[4] Tessone CJ, Sciré A, Toral R, Colet P (2007) Theory of collective firing induced by noise or diversity in excitable media. Phys Rev E 75: 016203. 10.1103/PhysRevE.75.016203 17358231

[5] Komin N, Murza A, García EH, Toral R (2010) Synchronization and entrainment of coupled circadian oscillators. Interface Focus 1: 167–176. 22419982 10.1098/rsfs.2010.0327 PMC3262239

[6] Astumian RD (1997) Thermodynamics and kinetics of a Brownian motor. Science 276: 917–922. 9139648 10.1126/science.276.5314.917

[7] Gassmann F (1997) Noise-induced chaos-order transitions. Phys Rev E 55: 2215–2221.

[8] Zaks M, Sailer X, Schimansky-Geier L, Neiman A (2005) Noise induced complexity: From subthreshold oscillations to spiking in coupled excitable systems. CHAOS 15: 026117. 10.1063/1.1886386 16035919

[9] Lindner B, García-Ojalvo J, Neiman A, Schimansky-Geier L (2004) Effects of noise in excitable systems. Phys Rep 392: 321–424.

[10] Wiesenfeld K, Moss F (1995) Stochastic resonance and the benefits of noise–from ice ages to crayfish and squids. Nature 373: 33–36. 7800036 10.1038/373033a0

[11] Gammaitoni L, Hänggi P, Jung P, Marchesoni F (1998) Stochastic resonance. Rev Mod Phys 70: 223–287.

[12] Longtin A (1993) Stochastic resonance in neuron models. J Stat Phys 70: 309–327.

[13] Braun H, Wissing H, Schafer K, Hirsch M (1994) Oscillations and noise determine signaltransduction in shark multimodal sensory cells. Nature 367: 270–273. 11407413 10.1038/367270a0

[14] Bezrukov S, Vodyanoy I (1998) Stochastic resonance in thermally activated reactions: Application to biological ion channels. CHAOS 8: 557–566. 12779759 10.1063/1.166337

[15] Longtin A (1997) Autonomous stochastic resonance in bursting neurons. Phys Rev E 55: 868–876.

[16] Chialvo D, Longtin A, Müller-Gerking J (1997) Stochastic resonance in models of neuronal ensembles. Phys Rev E 55: 1798–1808.

[17] Neiman A, Silchenko A, Anishchenko V, Schimansky-Geier L (1998) Stochastic resonance: Noiseenhanced phase coherence. Phys Rev E 58: 7118–7125.

[18] Postnova S, Voigt K, Braun HA (2009) A mathematical model of homeostatic regulation of sleepwake cycles by Hypocretin/Orexin. J Biol Rhythms 24: 523–535. 19926811 10.1177/0748730409346655

[19] Peyron C, Tighe DK, van den Pol AN, de Lecea L, Heller HC, et al (1998) Neurons containing hypocretin (orexin) project to multiple neuronal systems. J Neurosci 18: 9996–10015. 9822755 10.1523/JNEUROSCI.18-23-09996.1998 PMC6793310

[20] Yoshida K, McCormack S, España RA, Crocker A, Scammell TE (2006) Afferents to the orexin neurons of the rat brain. J Comp Neurol 494: 845–61. 16374809 10.1002/cne.20859 PMC2259441

[21] Winsky-Sommerer R, Yamanaka A, Diano S, Borok E, Roberts AJ, et al (2004) Interaction between the corticotropin-releasing factor system and hypocretins (orexins): A novel circuit mediating stress response. J Neurosci 24: 11439–11448. 15601950 10.1523/JNEUROSCI.3459-04.2004 PMC6730356

[22] Sakurai T (2007) The neural circuit of orexin (hypocretin): maintaining sleep and wakefulness. Nature Rev Neurosci 8: 171–81. 17299454 10.1038/nrn2092

[23] Li Y, Gao XB, Sakurai T, van den Pol AN (2002) Hypocretin/orexin excites hypocretin neurons via a local glutamate neuron–A potential mechanism for orchestrating the hypothalamic arousal system. Neuron 36: 1169–1181. 12495630 10.1016/s0896-6273(02)01132-7

[24] Borbély A (1982) A two-process model of sleep regulation. Hum Neurobiol 1: 195–204. 7185792

[25] Eggermann E, Bayer L, Serafin M (2003) The wake-promoting hypocretin-orexin neurons are in an intrinsic state of membrane depolarization. J Neurosci 23: 1557–62. 12629156 10.1523/JNEUROSCI.23-05-01557.2003 PMC6741978

[26] Borbély AA, Achermann P (2000) Sleep homeostasis and models of sleep regulation. In: Kryger M, Roth T, Dement WC, editors. Principles and practice of sleep medicine. Philadelphia: W.B. Saunders Company. pp. 377–390.

[27] Hodgkin AL, Huxley AF (1952) A quantitative description of membrane current and its application to conduction and excitation in nerve. J Physiol 117: 500–544. 12991237 10.1113/jphysiol.1952.sp004764 PMC1392413

[28] San Miguel M, Toral R (2000) Stochastic effects in physical systems. In: Tirapegui E, Martinez J, Tiemann R, editors. Instabilities and Nonequilibrium Structures VI. Dordrecht: Kluwer Academic Publishers, Nonlinear Phenomena and Complex Systems. pp. 35–130.

[29] Bazhenov M, Timofeev I, Steriade M, Sejnowski T (2002) Model of thalamocortical slow-wave sleep oscillations and transitions to activated states. J Neurosci 22: 8691–8704. 12351744 10.1523/JNEUROSCI.22-19-08691.2002 PMC6757797

[30] Hill S, Tononi G (2005) Modeling sleep and wakefulness in the thalamocortical system. J Neurophysiol 93: 1671–1698. 15537811 10.1152/jn.00915.2004

[31] Diniz Behn C, Brown E, Scammell T, Kopell N (2007) Mathematical model of network dynamics governing mouse sleep-wake behavior. J Neurophysiol 97: 3828–3840. 17409167 10.1152/jn.01184.2006 PMC2259448

[32] Best J, Diniz Behn C, Poe G, Booth V (2007) Neuronal models for sleep-wake regulation and synaptic reorganization in the sleeping hippocampus. J Biol Rhythms 22: 220–232. 17517912 10.1177/0748730407301239

[33] Diniz Behn C, Kopell N, Brown E, Mochizuki T, Scammell T (2008) Delayed orexin signalling consolidates wakefulness and sleep: physiology and modeling. J Neurophysiol 99: 3090–3103. 18417630 10.1152/jn.01243.2007 PMC3065358

[34] Fleshner M, Booth V, Forger D, Behn CD (2011) Circadian regulation of sleep–wake behaviour in nocturnal rats requires multiple signals from suprachiasmatic nucleus. Phil Trans R Soc A 369: 3855–3883. 21893532 10.1098/rsta.2011.0085

[35] Williams K, Diniz Behn C (2011) Dynamic interactions between orexin and dynorphin may delay onset of functional orexin effects: A modeling study. J Biol Rhythms 26: 171–181. 21454297 10.1177/0748730410395471

