# Supplementary material for: Diversity and Noise Effects in a Model of Homeostatic Regulation of the Sleep-Wake Cycle
Source: PLoS Comput Biol. 2012 Aug 23;8(8):e1002650. doi: 10.1371/journal.pcbi.1002650 (PMC3426568; doi:10.1371/journal.pcbi.1002650)
Supplement: Text S1 — Some details on the numerical simulation. The supporting Information file Text S1 contains some more detailed information about: A: the definition of the wakefulness time intervals during the day and the night, and , respectively, used for evaluating the quality of the sleep-wake cycle; B: the extraction procedure of the diversified threshold potentials and the form of the corresponding probability distribution . (PDF) [file pcbi.1002650.s001.pdf]

Text S1. Further details on the numerical simulation

A: Evaluation of the wake and sleep times

The quality of a sleep-wake cycle was estimates through the coefficient $r = \Delta t^{(1)}/\tau_1 - \Delta t^{(2)}/\tau_2$, where $\Delta t^{(\alpha)}$ represents the wakefulness time interval during the day ($\alpha = 1$) or night ($\alpha = 2$), while τ_1 and τ_2 represent the length of day and night, respectively. This coefficient can vary between a minimum value $r = -1$, representing an exchange of wakefulness and sleep between day and night, and a maximum value $r = 1$, corresponding to the optimal situation with wakefulness during the whole day time interval $\Delta t^{(1)} = \tau_1$ and continuous sleep during the whole night interval, $\Delta t^{(2)} = 0$. For a more realistic estimate of the quality of the sleep-wake cycle the two wakefulness time intervals $\Delta t^{(1)}$ and $\Delta t^{(2)}$ were computed in slightly different ways, as described below.

Estimating wakefulness during the day: $\Delta t^{(1)}$. In order to estimate the wakefulness during the day, when an individual is supposed to be awake, we have considered the quality of wakefulness by computing $\Delta t^{(1)}$ from states characterized by an "effective wakefulness" in a tonic firing regime. On the other hand, isolated spikes, which break a sleep period, did not contribute to the wakefulness interal and were neglected: such isolated spikes can at most represent a fragmented wakefulness state, similar to that of a narcoleptic individual. A tonic spiking state was recognized checking if the inter-spike time was smaller than a suitable threshold $\tau_{\rm max}$; in this case such an inter-spike interval was added to the total wakefulness time interval $\Delta t^{(1)}_{\rm wake}$. On the other hand, if the corresponding inter-spike time was larger than the threshold $\tau_{\rm max}$, then the two corresponding spikes were considered to be isolated ones and that time interval was neglected. Notice that in general inter-spike times are not universal and vary with external parameters; for this reason a value $\tau_{\rm max} = 100$ ms was chosen heuristically, considering the typical working conditions of the system.

Estimating wakefulness during the night: $\Delta t^{(2)}$. As for the night is concerned, it is the quality of sleep which contributes to the overall quality of the sleep-wake cycle. Therefore, on the difference of $\Delta t^{(1)}$, we have computed $\Delta t^{(2)}$, the wakefulness period during night, including also short wakefulness events (isolated spikes), since they break the sleep period, thus worsening the quality of sleep. Each isolated spike is assumed to contribute a conventional time interval, assumed as $\tau_{\rm max}$ for simplicity.

The difference between the two mentioned ways to evaluate wakefulness periods is relevant at high level of noise, which produces many isolated spikes.

B: Extraction of parameters with a given distribution

The different values of the glutamate thresholds $\{W_i\}$ of neurons $\{\mathrm{A}_i\}$, which make neurons A heterogeneous, were extracted using the probability distribution

$$f(W) = \frac{1}{2\,\delta W \cosh^2[(W - \bar{W})/\delta W]} \,. \tag{1}$$

Here $\bar{W} = \int dW\, W\, f(W)$ is the average value and the parameter δW measures the dispersion of the distribution around $\bar{W}$. It is proportional to the standard deviation σ, namely, it is related to the variance according to $\sigma^2 \equiv \langle (W - \bar{W})^2 \rangle = \pi^2 \delta W^2/12$. The function (1) can be integrated to obtain the lower cumulative distribution function $F(x)$,

$$F(W) = \frac{1}{2}\left\{1 + \tanh[(W - \bar{W})/\delta W]\right\} = \frac{1}{1 + \exp[-2(W - \bar{W})/\delta W]} \,. \tag{2}$$

Simulations were made by using various sets of A-neuron thresholds $\{W_i\}$ obtained by rescaling the values of a same set of thresholds by δW. This can be done for example by inverting the same uniform distribution of values $\{F_i\}$ and using distributions $F(W)$ corresponding to the different desired values of

δW. For all the parameter sets $\{W_i\}$ used the average value $\langle W \rangle$ was the same. For a few set of parameters we have checked that equivalent results are obtained by first extracting randomly the parameters from the distribution $F(x)$ with the desired δW and then averaging the final results obtained. In the latter case the set of values $\{F_i\}$ are extracted randomly in the interval $F_i \in (0, 1)$.
